# Third-generation cephalosporin resistant gram-negative bacteraemia in patients with haematological malignancy; an 11-year multi-centre retrospective study

**DOI:** 10.1186/s12941-022-00544-0

**Published:** 2022-11-28

**Authors:** Jara R. de la Court, Sjoukje H. S. Woudt, Annelot F. Schoffelen, Jarom Heijmans, Nick A. de Jonge, Tjomme van der Bruggen, Marije K. Bomers, Merel M. C. Lambregts, Rogier P. Schade, Kim C. E. Sigaloff, ISIS-AR study group, J. W. T. Cohen Stuart, D. C. Melles, K. van Dijk, A. Alzubaidy, B. F. M. Werdmuller, G. J. Blaauw, B. M. W. Diederen, H. J. Alblas, W. Altorf-van der Kuil, S. M. Bierman, S. C. de Greeff, S. R. Groenendijk, R. Hertroys, E. J. Kuijper, J. C. Monen, D. W. Notermans, W. J. van den Reek, A. E. Smilde, C. C. H. Wielders, R. E. Zoetigheid, W. van den Bijllaardt, E. M. Kraan, E. E. Mattsson, J. M. da Silva, E. de Jong, B. Maraha, G. J. van Asselt, A. Demeulemeester, B. B. Wintermans, M. van Trijp, A. Ott, J. Sinnige, D. C. Melles, W. Silvis, L. J. Bakker, J. W. Dorigo-Zetsma, K. Waar, A. T. Bernards, M. A. Leversteijn-van Hall, E. Schaftenaar, M. H. Nabuurs-Franssen, H. Wertheim, B. M. W. Diederen, L. Bode, M. van Rijn, S. Dinant, O. Pontesilli, P. de Man, M. Wong, A. E. Muller, N. H. Renders, R. G. Bentvelsen, A. G. M. Buiting, A. L. M. Vlek, A. J. Stam, A. Troelstra, I. T. M. A. Overdevest, M. P. A. van Meer, C. Oliveira dos Santos, M. J. H. M. Wolfhagen

**Affiliations:** 1 Department of Medical Microbiology and Infection Prevention, Amsterdam UMC, University of Amsterdam, Vrije Universiteit Amsterdam, Amsterdam, The Netherlands; 2 Division of Infectious Diseases, Department of Internal Medicine, Amsterdam UMC, University of Amsterdam, Vrije Universiteit Amsterdam, Amsterdam, The Netherlands; 3 Centre for Infectious Disease Control, National Institute for Public Health and the Environment, Bilthoven, The Netherlands; 4 Department of Haematology, Amsterdam UMC, University of Amsterdam, Vrije Universiteit Amsterdam, Amsterdam, The Netherlands; 5 Department of Infectious Diseases, Leiden University Medical Centre, Leiden University, Leiden, The Netherlands; 6 Department of Medical Microbiology, University Medical Centre Utrecht, University Utrecht, Utrecht, The Netherlands

**Keywords:** Febrile neutropenia, Antimicrobial resistance, Resistance surveillance, Empirical antibiotic therapy, Third-generation-cephalosporin resistance, Colonization.

## Abstract

**Objectives:**

Among patients with haematological malignancy, bacteraemia is a common complication during chemotherapy-induced neutropenia. Resistance of gram-negative bacteria (GNB) to third-generation cephalosporins (3GC) is increasing. In order to explore the value of using surveillance cultures to guide empirical treatment e.g. choosing between carbapenem versus ceftazidime- we aimed to assess the distribution of pathogens causing bacteraemia in patients with haematological malignancy, and the proportion of 3GC-resistant GNB (3GC-R GNB) bacteraemia that was preceded by 3GC-R GNB colonization.

**Methods:**

Using 11 years of data (2008–2018) from the Dutch national antimicrobial resistance surveillance system, we assessed the prevalence of 3GC-R GNB in episodes of bacteraemia, and the proportion of 3GC-R GNB bacteraemia that was preceded by 3GC-R GNB colonization. Colonization was defined as availability of any GNB surveillance isolate in the year before, independent of the causative micro-organism (time-paired isolates).

**Results:**

We included 3887 patients, representing 4142 episodes of bacteraemia. GNB were identified in 715/4142 (17.3%), of which 221 (30.9%) were 3GC-R GNB. In 139 of these 221 patients a time-paired surveillance culture was available. In 76.2% (106/139) of patients these surveillance cultures already showed 3GC-R GNB isolates in the year prior to the culture date of the 3GC-R GNB positive blood isolate.

**Conclusions:**

This multi-centre study shows that in patients with haematological malignancy, the majority of 3GC-R GNB bacteraemia is preceded by 3GC-R GNB colonization. Prospective clinical studies are needed to assess the safety and benefits of the use of surveillance-cultures to guide empirical therapy to restrict the empirical use of carbapenems in this population.

## Background

Fever frequently complicates chemotherapy-induced neutropenia in patients with haematological malignancy [1]. Half of febrile neutropenic (FN) episodes remain of unknown origin. In less than a quarter (20–23%) of FN episodes, blood cultures are positive for possible causative pathogens [2, 3]. Prompt initiation of antibiotics, with activity against important gram-negative bacteria (e.g. *Pseudomonas aeruginosa*), is critical in case of bloodstream infection [4–8]. An important source of bacteraemia in chemotherapy-induced FN is thought to be the gut [9]. Colonization with resistant bacteria is considered a major risk factor for bloodstream infection with resistant bacteria [10–13]. In Dutch hospitals treating high-risk neutropenic patients, surveillance cultures (e.g. on oral and rectal swabs) are performed to identify patients colonized with resistant microorganisms, and modify empirical antibiotic therapy accordingly. Apart from the Fourth European Conference on Infections in Leukaemia (ECIL-4) guideline, guidelines do not recommend to rely on surveillance cultures to guide the choice of empirical antibiotic therapy for FN, e.g. the choice between carbapenem versus ceftazidime [7, 8]. Prospective studies assessing the effectiveness of surveillance-culture-guided empirical therapy in patients with FN are scarce [14].

In order to provide rationale for surveillance-culture-guided empirical therapy for FN, we performed a multi-centre study among patients with haematological malignancy. Since 2008, data of Dutch medical microbiology laboratories are collected in the Infectious Diseases Surveillance Information System–Antimicrobial Resistance (ISIS-AR) [15]. In 2018, 47/54 (87%) laboratories were connected to ISIS-AR. Using ISIS-AR data, we aimed to assess the distribution of pathogens causing bacteraemia in patients with haematological malignancy, and the proportion of 3GC-R GNB bacteraemia that was preceded by 3GC-R GNB colonization.

## Methods

We performed a descriptive retrospective study of routine microbiological surveillance data collected through the Dutch national surveillance system for antimicrobial resistance over an 11-year period (2008–2018). ISIS-AR collects all positive microbiological culture results with antimicrobial susceptibility test derived using automated systems, gradient tests and/or the disk diffusion method, including minimum inhibitory concentration (MIC) values and inhibition zone diameters, of isolates routinely tested in medical microbiology laboratories in the Netherlands [15]. ISIS-AR contains data from university and non-university hospitals, as well as general practices and long-term care facilities. Basic patient information such as age, sex and hospital ward is available, but does not include clinical variables (e.g. diagnosis, comorbidities, and treatment regimens). ISIS-AR does not contain data about negative culture results, or about cultured isolates without phenotypical antimicrobial susceptibility test.

Data was extracted on positive cultures with a sampling date between 2008 and 2018 from both adult and paediatric patients admitted to a haematology ward at the date of sampling or for whom the culture was requested by a haematologist. As the sole indication to obtain surveillance cultures on the haematology ward in the Netherlands is the weekly assessment of colonization status in patients receiving high-risk chemotherapy (myelosuppressive chemotherapy resulting in myelosuppression and mucositis), we used surveillance samples gathered on the haematology ward as a proxy to identify patients that received high-risk chemotherapy [16]. In some hospitals surveillance cultures are also used to identify colonization with other pathogenic micro-organisms (e.g. *Candida* species). As surveillance cultures are also obtained on the Dutch Intensive Care units (ICUs) in non-haematology patients we excluded culture from ICU patients. Because most high-risk neutropenic patients are treated in-hospital we also excluded cultures from outpatients. We extracted data on isolates taken for non-diagnostic, specified as such by the laboratory, often based on (a combination of) specimen type, specialty and specific culture requests (e.g. targeted screening for resistant microorganisms). These isolates, the majority consisting of rectal or pharyngeal/upper airway tract cultures, are further referred to as surveillance isolates and considered a proxy for colonization status. We also extracted data on diagnostic blood isolates submitted only by those laboratories for which data on surveillance cultures were available at any time between 2008 and 2018. We categorized all isolates as Gram-positive, Gram-negative or not applicable/unknown and by (group of) microorganism based on clinical and/or microbiological relevance. Isolates of GNB were categorized by their susceptibility to third-generation cephalosporins (3GC) and carbapenems, after reinterpretation of MIC values according to clinical breakpoints set by the European Committee on Antimicrobial Susceptibility Testing (EUCAST, version 8.0; 2018), and prioritizing results from gradient tests over automated MIC results. Zone diameter values were excluded, as methods for disk diffusion are not harmonized across laboratories, changed over time, and exact inhibition zone diameters are often not reported to ISIS-AR. For microorganisms without EUCAST breakpoints, or isolates without MIC values, the S/I/R interpretation as reported by the laboratory was used, if available. A GNB isolate was categorized as 3GC-R if intermediately susceptible or resistant (I + R) to ceftriaxone, cefotaxime or ceftazidime, and otherwise as 3GC-S. GNB isolates without susceptibility testing for any of these antimicrobials were excluded from the analysis. Enterobacterales species with constitutive AmpC production -e.g. *Citrobacter freundii*, *Enterobacter cloacae complex*, *Klebsiella aerogenes* and *Serratia marcescens*- were regarded as intrinsically resistant and labelled as 3GC-R regardless of MIC, since selection of AmpC de-repressed cephalosporin-resistant mutants can occur during therapy. *Stenotrophomonas maltophilia* isolates were also categorized as 3GC-R, regardless of MIC [17]. GNB were categorized carbapenem-resistant if intermediately susceptible or resistant (I + R) to meropenem or imipenem, and otherwise as non-carbapenem-resistant (i.e. carbapenem-susceptible or unknown).

We performed two analyses to answer two research questions. In analysis 1, to assess the distribution of microorganisms and the prevalence of 3GC-R GNB in bacteraemia, we approximated a representative sample of bacteraemia episodes, in different stages of treatment, by including the first blood isolate—regardless of the microorganism or its resistance profile- within a period of six months for each patient. Herewith we aimed to avoid oversampling of patients with recurrent or persistent infections and provide an unbiased distribution of pathogens causing bacteraemia in this patient population. We calculated the distribution of (groups of) microorganisms and the proportion of 3GC-R GNB found during bacteraemia. Among isolates of 3GC-R GNB, we counted specific species, and the number of carbapenem-resistant isolates. In analysis 2, to determine the proportion of 3GC-R GNB bacteraemia preceded by 3GC-R GNB colonization, we combined data on all surveillance and blood isolates per patient. In this combined dataset, we identified patients with paired GNB surveillance and blood isolates, defined as patients having a GNB surveillance isolate, followed by a GNB blood isolate in the 3–365 days thereafter. This pair was made independent of exact causative micro-organism, since colonization with any 3GC-R GNB pathogen could result in a change of antibiotic management. If multiple isolates per patient were available, either from the same or from different cultures, we prioritized 3GC-R GNB over 3GC-S GNB. If multiple pairs were eligible for a patient we selected the pair of isolates with the shortest time interval. Patients with a paired surveillance and blood isolate were categorized according to 3GC susceptibility of both isolates: neither 3GC-R, either the surveillance isolate or blood isolate 3GC-R, or both 3GC-R. Among patients with a 3GC-R GNB blood isolate and a paired GNB surveillance isolate, we calculated the median number of days between both isolates. To assess the influence of the maximum time interval—between a paired surveillance and blood isolate (365 days)—on the proportion of 3GC-R GNB bacteraemia preceded by 3GC-R GNB colonization, we performed a sensitivity analysis using a 30-day maximum interval. In identifying patients with paired surveillance and blood isolates, we selected patients with a paired GNB surveillance and GNB blood isolate, rather than patients with any reported surveillance isolate regardless of the microorganism and a GNB blood isolate. Due to selective culture methods used by most laboratories to detect colonization with GNB (or other pathogenic microorganisms, varying between laboratories), and since cultures without antimicrobial susceptibility test results are not available in the ISIS-AR database, the total number of reported surveillance isolates is an underestimation of the true number of colonized patients and was therefore not used in this analysis.

All analyses were performed using SAS software (version 9.4; SAS Institute, Cary, North Carolina). As no identifiable personal data are collected, individual patient consent was not required for this study.

## Results

Between 2008 and 2018, 12,191 blood isolates from 3887 patients and 21,383 surveillance isolates from 4790 patients were available in the ISIS-AR database. These data were submitted by eleven laboratories representing 13 hospitals. Of 3887 patients with bacteraemia, 3074 (79.0%) were treated in tertiary care or specialized hospitals.

### Distribution of pathogens and proportion of 3GC-R GNB in blood isolates

**Fig. 1:**
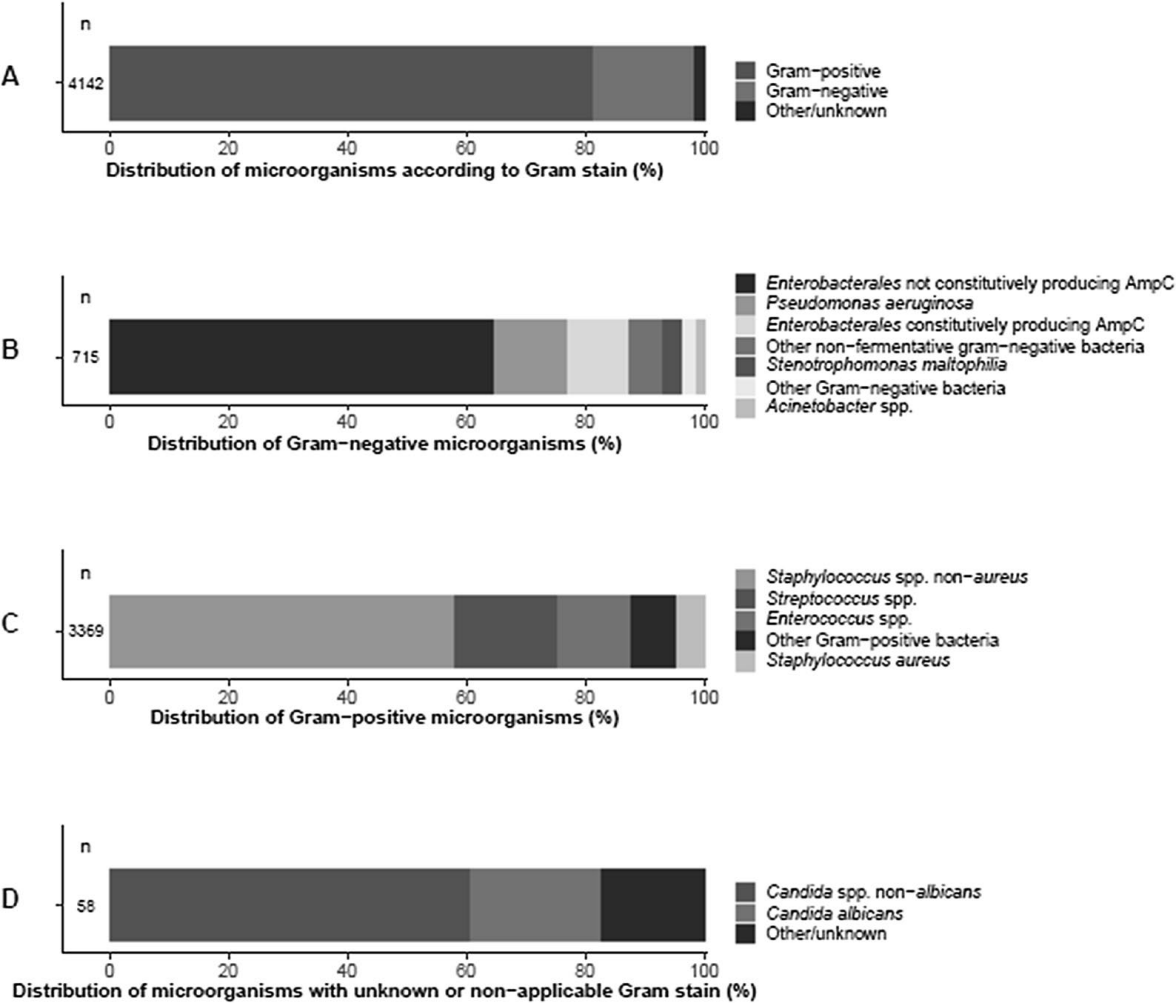
Distribution of (groups of) microorganisms by Gram-stain (**A**), among Gram-negative bacteria (**B**), among Gram-positive bacteria **C** and among microorganisms with unknown or non-applicable Gram-stain **D** in 4142 episodes of bacteraemia among 3887 patients, 2008–2018

In total, 4142 of 12,191 blood isolates were identified and included as representative of an episode of bacteraemia. The majority of isolates (3369, 81.3%) comprised Gram-positive bacteria, 715 (17.3%) were Gram-negative bacteria, and for 58 isolates (1.4%) Gram stain categorization was not possible (e.g. due to incomplete microorganism identification) or not applicable (Fig. 1A). Among Gram-negative bacteria, Enterobacterales species not constitutively producing AmpC were most frequently isolated (464, 64.9% of GNB, 11.2% of the bacteraemia episodes), whereas *P. aeruginosa* (86, 12.0% of GNB, 2.1% of the bacteraemia episodes) and Enterobacterales species constitutively producing AmpC (76, 10.6% of GNB, 1.8% of the bacteraemia episodes) were less frequently found (Table 1, Fig. 1B). Among Gram-positive species, *Staphylococcus* spp. non-*aureus* (i.e. mainly coagulase-negative staphylococci) comprised the majority of isolates (1957, 58.1%), followed by *Streptococcus* spp*.* (574, 17.0%). *Staphylococcus aureus* was less frequently isolated (149, 4.4%, Fig. 1C). *Candida* species were found in 48 blood isolates (82.8% of isolates with unknown or non-applicable Gram stain, 1.2% of the bacteraemia episodes, Fig. 1D).

**Table 1:** Third-generation cephalosporin-resistant (3GC-R) and carbapenem-resistant Gram-negative bacteria isolated from 4142 episodes of bacteraemia in 3887 patients with haematological malignancies, 2008–2018

	Total	3GCR, N	Carbapenem-resistant among 3GCR, N
Enterobacterales not constitutively producing AmpC	464	85	1
- *Escherichia coli*	342	58	0
- *Klebsiella pneumoniae*	80	25	1
- Other*	42	3	0
Enterobacterales constitutively producing AmpC**	76	76	0
- *Citrobacter freundii*	8	8	0
- *Enterobacter* species	37	37	0
- *Klebsiella aerogenes*	6	6	0
- *Serratia* species	22	22	0
- *Morganella* species	3	3	0
*Pseudomonas aeruginosa*	86	8	5
*Acinetobacter* species	10	7	0
Stenotrophomonas maltophilia***	22	22	22
Other non-fermentative gram-negative bacteria	39	15	9
Other gram-negative bacteria	18	8	1
Total	715	221	38

*Other Enterobacterales not constitutively producing AmpC were: *Citrobacter* species (non-*freundii*), *Pantoea* species, *Proteus* species and *Salmonella* species

**Enterobacterales constitutively producing AmpC *(e.g. Citrobacter freundii, Enterobacter cloacae complex, Klebsiella aerogenes and Serratia marcescens)* were regarded as intrinsically resistant and therefore labelled as 3GC-R regardless of MIC

***Stenotrophomonas maltophilia was categorized as 3GC-R and carbapenem resistant, regardless of MIC

In total, 221 isolates were 3GC-R GNB, which is 30.9% of GNB isolates and 5.3% of the bacteraemia episodes. The most commonly isolated species among 3GC-R GNB were Enterobacterales not constitutively producing AmpC (85, 38.5%) and Enterobacterales constitutively producing AmpC (76, 34.4%, Table 1). The proportion of 3GC-R among Enterobacterale*s* not constitutively producing AmpC, i.e. likely extended-spectrum beta-lactamase (ESBL)-producing Enterobacterales, was 18.3% (85/464, Table 1). Carbapenem resistance was present in 38/221 (17.2%) isolates (Table 1), the majority (57.9%) due to 22 *S. maltophilia* isolates regarded as intrinsically resistant to beta-lactam antibiotics. Five out of eight 3GC-R *P. aeruginosa* isolates were also resistant to carbapenems.

### Paired GNB surveillance and blood isolates

**Fig. 2:**
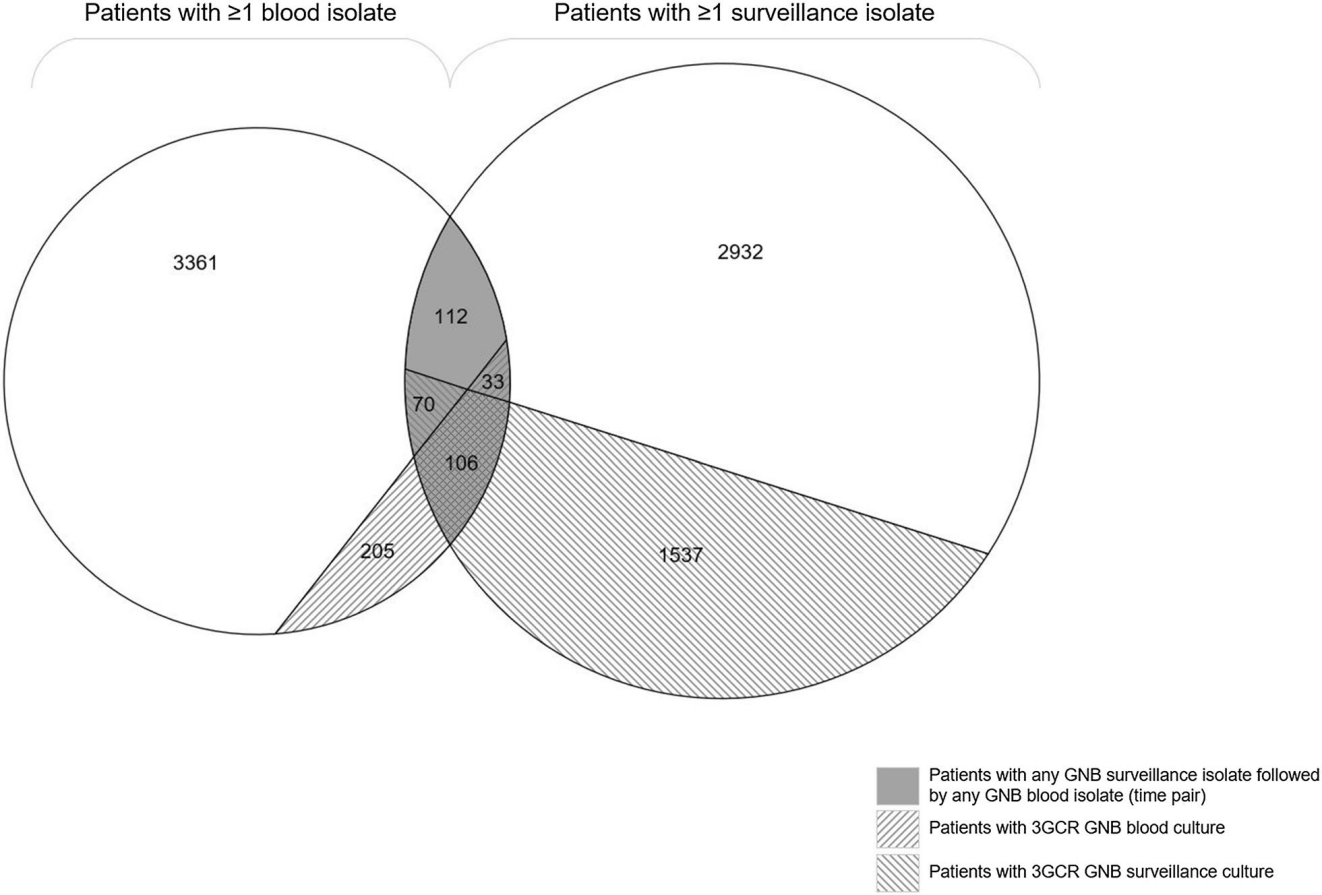
Schematic overview^1^ of numbers of patients with (3GC-R GNB) blood and surveillance isolates, and patients with any GNB surveillance isolate followed by any GNB blood culture within 1 year (time pair) between 2008 and 2018. In total, 321 patients with time-paired isolates were identified, represented by the grey overlay. In 139 patients with 3GC-R GNB blood isolates a time-paired surveillance isolate was available, represented with the slash up to the right. In 106/139 the 3GC-R GNB blood isolates was preceded by a 3GC-R GNB surveillance isolate, represented by the crossed lines. ^1^The overview is included for illustrative purposes, the proportions displayed in this diagram are only an approximation of true proportions. *3GC-R* third-generation cephalosporin-resistant. *GNB* Gram-negative bacteria

Paired GNB surveillance and blood isolates, i.e. *any* GNB surveillance isolate, followed by *any* GNB blood isolate, were identified for 321 patients (Fig. 2), of which 43.3% (139/321) had a 3GC-R GNB blood isolate. In those 139 patients with 3GC-R GNB blood isolate, the blood isolate was found after a median of 10 days (IQR 5–23 days) after the GNB surveillance isolate. In 76.2% (106/139), the 3GC-R GNB blood isolate was preceded by a GNB surveillance isolate that was also 3GC-R. When reducing the maximum interval between the paired isolates to 30 days instead of 365 days in a sensitivity analysis, the percentage of 3GC-R GNB bacteraemia preceded by 3GC-R GNB surveillance cultures was similar (75.4%, 86/114). In 54.8% (176/321) of the patients with paired isolates, a 3GC-R GNB surveillance isolate was present.

## Discussion

In this multi-centre study among patients with haematological malignancy admitted to Dutch hospitals -in the past decade- the overall proportion of 3GC-R GNB bacteraemia was 5.3%. In a subset of 321 patients with paired isolates -patients with a GNB surveillance isolate followed by a GNB blood isolate within a year- 76.2% of 3GC-R GNB bacteraemia was preceded by 3GC-R GNB colonization as identified using surveillance cultures.

In the first analysis, based on a representative sample of bacteraemia episodes in different stages of treatment of malignancy, 81.3% of blood isolates were Gram-positive isolates. As the etiological relevance of these species could not be determined, contamination rather than bacteraemia could not be ruled out. Nonetheless, Gram-negative bacteria were retrieved in a minority (17.3%) of positive blood cultures, and etiological relevance of these bacteria as causative agents of bacteraemia is not debated. Interestingly, 18.3% of Enterobacterales not constitutively producing AmpC was 3GC-R. This is higher than the ≤ 9% observed among *E. coli, K. pneumoniae* and *P. mirabilis* in the general inpatient population in the Netherlands in 2019 and the 4% ESBL *Escherichia coli* found in a survey in 2003–2010 in France [18, 19]. The substantially higher GNB resistance rate found in our study is likely explained by high prophylactic and therapeutic antimicrobial consumption in this population, compared to the general inpatient population [20, 21]. In our study, 30.9% of all GNB blood isolates was 3GC-R GNB which is similar to the prevalence of 27.6% among hematopoietic stem cell transplantation recipients in northwest Europe [22]. The prevalence of 3GC-R GNB is significantly higher in southeast Europe, with rates up to 47% in an Italian study [23], which corresponds to the median of 43% 3GC-R GNB bacteraemia among adult haematology and cancer patients worldwide [24]. Of note, the definition used for 3GC-R GNB in these studies is based on susceptibility testing of GNB and does not include intrinsically resistant GNB. Therefore, resistance is expected to be even higher if the definition of 3GC-R GNB used in our study would be applied.

Our finding that 76.2% of 3GC-R GNB bacteraemia was preceded by 3GC-R GNB colonization, is in line with previous studies. [10–12, 25–28] In these studies, the sensitivity of colonization with multidrug-resistant (MDR) bacteria for MDR blood stream infection ranged from 45 to 91%. For ESBL-producing *Enterobacterales* (ESBL-E) bacteraemia, 73.9–99.8% were preceded by ESBL-E colonization, indicating that bacteraemia in the absence of known colonization is uncommon [11, 12, 25–27].

While the high resistance rates found in GNB in our study can be used to justify carbapenem usage for all haematological patients with FN. This carbapenem for all approach has many disadvantages. First, carbapenems are last-resort antibiotics and using them for all patients with FN—the majority with negative blood cultures and in case of positive blood cultures only a minority is positive with 3GC-R GNB isolates (5.3% of bacteraemia episodes in our study)—could potentially induce or select multidrug-resistant bacteria. Second, the use of carbapenems is associated with the predisposition to fungal infections and development of *Clostridium difficile*-associated diarrhoea [2, 3, 29–32]. Our study illustrates that the value of a surveillance-culture-guided approach, to restrict carbapenems for patients colonized with 3GC-R GNB, is that 76.2% (106/139) of 3GC-R GNB bacteraemia are anticipated by surveillance cultures and would therefore receive appropriate treatment. This approach thereby reduces the risk of inappropriately treated 3GC-R GNB bacteraemia without the necessity to administer carbapenems to all high-risk neutropenic patients with fever. Considering patients with paired isolates, 139/321 (43.3%) patients with 3GC-R GNB bacteraemia would have received inappropriate empirical antimicrobial therapy if ceftazidime was the standard. With surveillance-culture-guided empirical therapy this number can be reduced to 33/321 (10.3%), given the fact that in 106/139 (76.2%) patients 3GC-R GNB colonization had been identified. Considering that 20–23% of blood cultures during FN episodes are positive for possible causative pathogens [2, 3], and that, in our study, 17.3% of these blood cultures are positive for GNB, this would result in 0.4% inappropriate empirical antimicrobial therapy per FN episode. At the same time, 145/321 (47.2%) patients were not colonized with 3GC-R GNB, and would receive ceftazidime in a surveillance-culture guided approach, substantially reducing carbapenem usage compared to an empirical regimen with standard carbapenem for all patients with FN.

This multi-centre study allowed us, for the first time in the Netherlands, to identify the distribution of pathogens in bacteraemia among patients admitted to haematology wards. The large and complete microbiological dataset reflects routine clinical practice as all consecutive surveillance and blood isolates with antimicrobial susceptibility tests were available. In this large dataset with structured and complete data on both surveillance and blood isolates, we were also able to identify patients in which bacteraemia was preceded by colonization. However, this study also has limitations. Due to limited clinical data we had to use a proxy to identify patients that received high-risk chemotherapy. Therefore the results are only representative of the inpatient haematology population. Furthermore, we only assessed concordance between surveillance and blood isolates in patients in whom GNB were isolated in both cultures, thus narrowing the scope of these results. However, because surveillance cultures are performed routinely in high-risk neutropenic patients in the Netherlands, this scope is of interest as it reflects this specific patient population. The time interval of one year between paired GNB isolates was based upon ESBL-carriage in travellers in whom most (88.7%) decolonized within one year [33]. This choice did not influence the outcome, as our sensitivity analysis shortening the interval to one month yielded similar results.

## Conclusion

In this study, we found a high 3GC-R GNB bacteraemia prevalence in the haematology population compared to the general in hospital population. The majority of these 3GC-R GNB bacteraemia are preceded by 3GC-R GNB colonization. Therefore, in centres in which routine surveillance cultures are performed, a surveillance-culture guided empirical therapy could be a viable strategy to restrict the empirical use of carbapenems in this population. However, prospective clinical studies are needed to further assess the safety and benefits of this approach.

## Data Availability

We will share aggregated, anonymized data, which have been used for this publication, upon reasonable request.
